# Hairy Cell Leukemia Masquerading as Pancytopenia in Pregnancy

**DOI:** 10.1155/2019/3238168

**Published:** 2019-08-21

**Authors:** Louisa Shackleton, Stephen E. Langabeer, David O'Brien, Sarah L. McCarron, Bridgette Byrne, Rupert Barry, Richard Flavin, C. Larry Bacon, Catherine M. Flynn

**Affiliations:** ^1^Department of Haematology, St. James's Hospital, Dublin 8, Ireland; ^2^Cancer Molecular Diagnostics, St. James's Hospital, Dublin 8, Ireland; ^3^Maternal Medicine Service, Coombe Women and Infants University Hospital, Dublin 8, Ireland; ^4^Department of Dermatology, St. James's Hospital, Dublin 8, Ireland; ^5^Department of Histopathology, St. James's Hospital, Dublin 8, Ireland

## Abstract

Thrombocytopenia is one of the most common hematological abnormalities observed during pregnancy, and in rare cases, this may be the first indicator of an underlying hematological malignancy. Hairy cell leukemia (HCL) is an uncommon B-cell lymphoproliferative disorder of which thrombocytopenia is a recurrent presenting feature. A case of pancytopenia presenting in pregnancy is described in which the thrombocytopenia persisted postpartum coincidental with a vesicular, pustular rash characterised as Sweet's syndrome. Hematological, histological, immunophenotypic, and molecular investigations confirmed the presence of HCL. The patient was treated with cladribine resulting in resolution of Sweet's syndrome, hematological remission from HCL, and achievement of a normal platelet count. This case highlights the need to maintain a wide differential diagnosis for presentations of pancytopenia or thrombocytopenia in pregnancy and the requirement for follow-up investigation of unusual cases with a lack of response to steroids or immunoglobulin.

## 1. Introduction

Thrombocytopenia develops in up to 10% of women during pregnancy or in the immediate postpartum period with approximately three quarters of cases attributed to gestational thrombocytopenia where the thrombocytopenia is mild and does not necessitate active management [1, 2]. Immune thrombocytopenia (ITP) is the most common cause in the first trimester but only accounts for approximately 4% of cases. Recent evidence suggest that in those patients with a platelet count less than 100 × 10^9^/L, a cause other than pregnancy or its complications should be considered [3]. In rare cases, the thrombocytopenia may be the first presenting feature of a hematological malignancy with this incidence ranging from one in one thousand to one in ten thousand pregnancies; subsequent management remains a challenging endeavour [4]. Of those hematological malignancies reported during pregnancy, hairy cell leukemia (HCL) is exceedingly rare [5, 6].

HCL is an uncommon B-cell lymphoproliferative neoplasm usually presenting in the sixth decade with pancytopenia and splenomegaly and which is five times more common in men than women [7]. Morphologically, hairy cells possess distinct cytoplasmic projections and by immnophenotyping characteristically express CD11c, CD25, and CD103 in addition to pan-B cell antigens. The discovery of the *BRAF* V600E mutation in nearly all cases of classical HCL has led to the introduction of targeted agents in those cases refractory to standard therapies of purine nucleoside analogues [8, 9]. An unusual case of thrombocytopenia in pregnancy leading to a subsequent postpartum diagnosis of HCL is presented.

## 2. Case Report

A 37-year-old woman was noted to have pancytopenia on routine blood screening during the first trimester of pregnancy. Complete blood count demonstrated a platelet count of 85 × 10^9^/L, white cell count of 2.3 × 10^9^/L (of which neutrophils were 1.3 × 10^9^/L and monocytes were 0.2 × 10^9^/L), and hemoglobin 10.6 g/dL. She reported occasional easy bruising in the last two months with no other bleeding issues, lymphadenopathy, or splenomegaly. The patient had one previous uncomplicated pregnancy delivered thirteen months previously with normal platelet counts throughout and had been in good health prior to this pregnancy. She had a personal history of equivocal hypothyroidism, had a family history of hypothyroidism and systemic lupus erythematosus, was on no regular medications, and had not commenced any new medications in pregnancy. Thyroid function tests, immunoglobulins, and folate and vitamin B_12_ levels were within normal range with a negative antinuclear antibody test. As a low serum ferritin level was noted, she commenced on iron supplementation. The patient was given a presumptive diagnosis of immune thrombocytopenia in pregnancy. During pregnancy, fatigue was her only symptom and she had no bleeding complications with her platelet count remaining static. There were no clinical findings to suggest preeclampsia or HELLP (hemolysis, elevated liver enzymes, and low platelet count) syndrome. Blood film examination was performed routinely throughout pregnancy with no morphological abnormalities observed. At 38-week gestation (platelets 61 × 10^9^/L), she was commenced on prednisolone 20 mg oral daily, but the platelet count showed no response (platelets 65 × 10^9^/L) prompting immunoglobulin therapy at 1 g/Kg of booking weight (70 g once-only dose), but this again had no effect on the platelet count (platelets 52 × 10^9^/L). Soon after, she presented to a maternity hospital in spontaneous labor and had a normal vaginal delivery at full term with minor bleeding and no postpartum hemorrhage. The platelet count was 52 × 10^9^/L at the time of delivery, and no therapy was given. Her male infant had a normal platelet count at birth.

**Figure 1:**
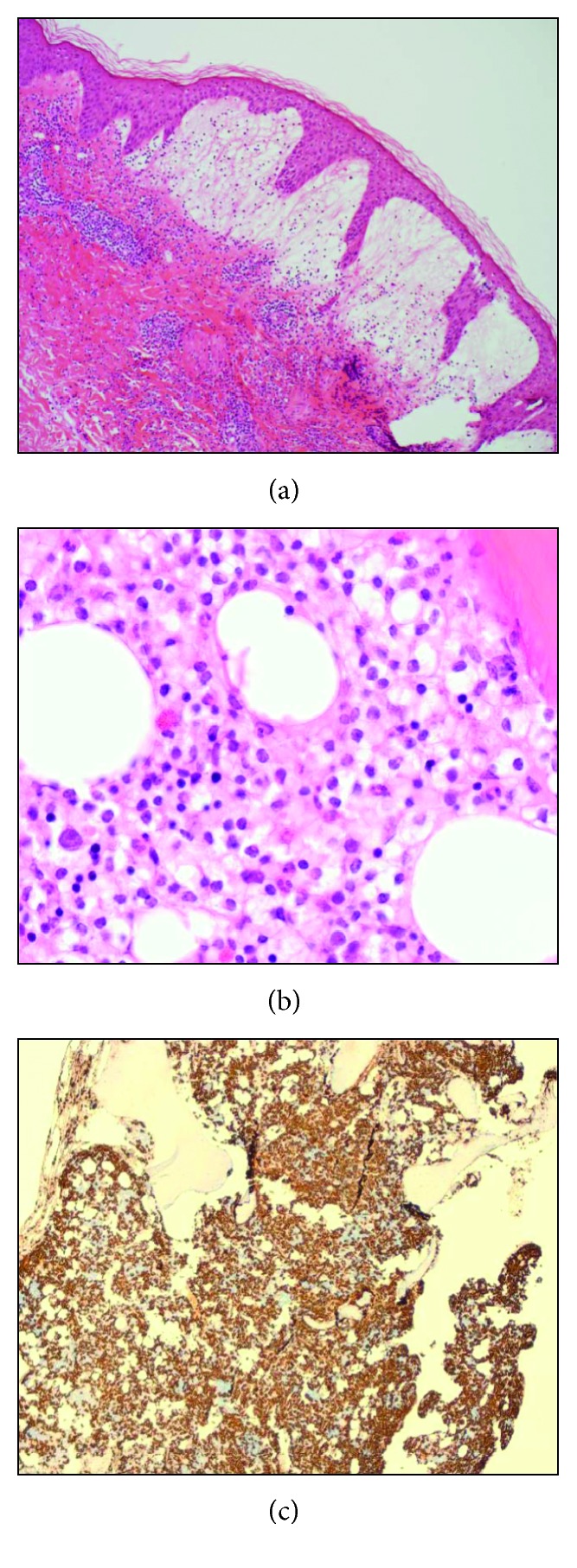
(a) Dermal infiltration of neutrophils of Sweet's syndrome, (b) bone marrow trephine demonstrating hairy cell leukemia infiltration, and (c) bone marrow trephine immunohistochemistry for CD20.

At review five weeks postpartum, the patient had developed a pustular skin rash on her left upper arm, lower abdomen, and lower back. A skin biopsy demonstrated dermal neutrophils producing upper dermal edema and a subepidermal blister consistent with bullous Sweet's syndrome (acute febrile neutrophilic dermatosis) (Figure 1(a)). Her hematological indices displayed persistent cytopenias with a hemoglobin of 13.0 g/dL, a white cell count of 1.7 × 10^9^/L (of which neutrophils were 0.5 × 10^9^/L and monocytes were 0.1 × 10^9^/L), and a platelet count of 49 × 10^9^/L. Occasional hairy cells were noted for the first time on blood film examination. Immunophenotyping demonstrated a population of B lymphocytes that represented 5% of non-erythroid cells. These B cells were kappa-restricted and positive for CD10, CD11c, CD19, CD20, CD25, and CD103. The bone marrow aspirate was normocellular for age and showed 23% lymphocytes with 10% having hairy projections. Trephine biopsy demonstrated an infiltration of hairy cells with typical morphology (Figure 1(b)) with immunohistochemistry of these hairy cells demonstrating positivity for CD10, CD20, CD25, CD72, and *BCL*2 (Figure 1(c)). The *BRAF* c.1799T > *A*; p.(Val600Glu) mutation was detected in genomic DNA from the bone marrow by a next-generation sequencing approach; all of which were consistent with a diagnosis of HCL.

Following this diagnosis of HCL, the patient received a single five-day course of cladribine at a dose of 0.1 mg/kg/day by continuous intravenous infusion at nine weeks postpartum. This was complicated by an episode of febrile neutropenia and a diffuse erythematous rash secondary to allopurinol and co-trimoxazole which resolved following cessation of these medications. At the last follow-up seven months after cladribine treatment, the patient is well with hemoglobin of 13.1 g/dL, a white cell count of 4.2 × 10^9^/L (of which neutrophils were 2.9 × 10^9^/L and monocytes were 0.3 × 10^9^/L), and platelet count of 207 × 10^9^/L with resolution of Sweet's syndrome. At this center, in such a case of favorable dermatological and hematological response, repeat bone marrow analysis to assess immunophenotypic or molecular residual disease is not routinely performed unless clinically indicated.

## 3. Discussion

The family history of autoimmune disease for this patient suggested a diagnosis of ITP that can occur in the first or early second trimester of pregnancy and in which the platelet count does not necessarily improve postpartum, unlike gestational thrombocytopenia. However, the absence of thrombocytopenia in a previous pregnancy and, more tellingly, the lack of response to steroids and immunoglobulins were the atypical, discordant clinical features suggestive of an alternative etiology.

This patient's presentation is unusual in its association of a vesicular eruption consistent with bullous Sweet's syndrome with HCL. Sweet's syndrome is a neutrophilic dermatosis with a known association with hematological malignancies particularly acute myeloid leukemia [10] but rarely associated with HCL [11–13]. Consistent with another case, the patient's rash in this case resolved following chemotherapy [13].

HCL is uncommon in women of reproductive age and has only been sporadically reported during pregnancy [14]. In such instances, successful treatment has been documented with interferon-alpha, rituximab, purine analogues, and laparoscopic splenectomy [14–16]. However, given the rarity of this presentation, no definitive treatment guideline has been possible with more information needed to establish the best management of HCL in pregnancy.

This case highlights the need to maintain a wide differential diagnosis for presentations of pancytopenia and thrombocytopenia in pregnancy and for reevaluation of persistent thrombocytopenia postpartum particularly when unresponsive to standard treatments.

## References

[1] Cines D. B., Levine L. D. (2017). Thrombocytopenia in pregnancy. *Blood*.

[2] Fogerty A. E. (2018). Thrombocytopenia in pregnancy: mechanisms and management. *Transfusion Medicine Reviews*.

[3] Reese J. A., Peck J. D., Deschamps D. R. (2018). Platelet counts during pregnancy. *New England Journal of Medicine*.

[4] Rizack T., Mega A., Legare R., Castillo J. (2009). Management of hematological malignancies during pregnancy. *American Journal of Hematology*.

[5] Williams J. K. (1987). Hairy cell leukemia in pregnancy: a case report. *American Journal of Obstetrics and Gynecology*.

[6] Pastner B., Penney R. W., Walsh C. M. (1994). Recurrent hairy cell leukemia during pregnancy: a case report. *American Journal of Obstetrics and Gynecology*.

[7] Kreitman R. J., Arons E. (2018). Update on hairy cell leukemia. *Clinical Advances in Hematology & Oncology*.

[8] Falini B., Martelli M. P., Tiacci E. (2016). *BRAF*V600E mutation in hairy cell leukemia: from bench to bedside. *Blood*.

[9] Grever M. R., Abdel-Wahab O., Andritsos L. A. (2017). Consensus guidelines for the diagnosis and management of patients with classic hairy cell leukemia. *Blood*.

[10] Kazmi S. M., Pemmaraju N., Patel K. P. (2015). Characteristics of sweet syndrome in patients with acute myeloid leukemia. *Clinical Lymphoma Myeloma and Leukemia*.

[11] Kramers C., Raemaekers J. M. M., van Baar H. M. J., de Pauw B. E., Horrevorts A. M. (1992). Sweet’s syndrome as the presenting symptom of hairy cell leukemia with concomitant infection by mycobacterium kansasii. *Annals of Hematology*.

[12] Ventura F., Rocha J., Pereira T., Herlander M., Fernando P., Celeste B. (2009). Sweet syndrome as the presenting symptom of hairy cell leukaemia. *Dermatology Online Journal*.

[13] Alkayen M., Cheng W. (2014). A case report of hairy cell leukemia presenting concomitantly with sweet syndrome. *Case Reports in Medicine*.

[14] Daver N., Nazha A., Kantarjian H. M., Haltom R., Ravandi F. (2013). Treatment of hairy cell leukemia during pregnancy: are purine analogues and rituximab viable therapeutic options. *Clinical Lymphoma Myeloma and Leukemia*.

[15] Baer M. R., Ozer H., Foon K. A. (1992). Interferon-*α* therapy during pregnancy in chronic myelogenous leukaemia and hairy cell leukaemia. *British Journal of Haematology*.

[16] Adeniji B. A., Fallas M., Incerpi M., Hamburg S., Katz R., Ogunyemi D. (2010). Laparoscopic splenectomy for hairy cell leukemia in pregnancy. *Case Reports in Medicine*.

